# Integrated clinical experience with concurrent problem-based learning is associated with improved clinical reasoning among physical therapy students in the United States

**DOI:** 10.3352/jeehp.2018.15.30

**Published:** 2018-12-25

**Authors:** Brad Warren Willis, Anita Sethi Campbell, Stephen Paul Sayers, Kyle Gibson

**Affiliations:** Department of Physical Therapy, School of Health Professions, University of Missouri, Columbia, MO, USA; Hallym University, Korea

**Keywords:** Clinical reasoning, Integrated clinical experience, Physical therapy, Problem-based learning, United States

## Abstract

Clinical reasoning (CR) is a key learning domain for physical therapy educators and a core skill for entry-level practitioners. Integrated clinical experience (ICE) and problem-based learning (PBL) have each been reported to improve interpersonal and social domains, while promoting knowledge acquisition and CR. Unfortunately, studies monitoring CR during ICE with concurrent PBL in physical therapy education are sparse. We hypothesized that ICE with concurrent PBL would be associated with improved self-reported CR in third-year student physical therapists (PTs) in the United States. The Self-Assessment of Clinical Reflection and Reasoning (SACRR) survey was administered to 42 student PTs at the beginning and end of their third and final year of didactic training. Between the pretest and posttest analyses, the participants completed faculty-led ICE and PBL coursework for 16 weeks. The overall SACRR score and 26 individual item scores were examined. The Wilcoxon rank-sum test and paired t-test were used, with statistical significance accepted at P< 0.05. Significant improvements were observed in the overall SACRR score (P< 0.001), including 6 of the 26 survey items centered around decision-making based on experience and evidence, as well as self-reflection and reasoning. ICE with PBL was associated with improved self-assessed CR and reflection in third-year student PTs in the United States. Monitoring the impact of curricular design on CR may improve educators’ ability to enhance cognitive and psychomotor skills, which underscores the importance of increasing the explicit use of theoretical frameworks and teaching techniques for coping with uncertainty as a way of enhancing entry-level training.

The aim to develop clinical reasoning (CR) in medicine and allied health trainees is resounding, particularly as health care becomes increasingly multifaceted across dynamic clinical practice settings [1,2]. Specifically, physical therapy educators have identified CR development in entry-level practitioners to be a key learning domain and central component of professional accreditation [1]. While multiple definitions exist, CR has been defined as “a complex cognitive process that uses formal and informal thinking strategies to gather and analyze patient information, evaluate the significance of this information and determine the value of alternative actions” [2]. As such, the integration of longitudinal CR assessments to elucidate the impact of various classroom and clinical curricular designs on this vital skill has been suggested [3].

Although integrated clinical experience (ICE) promoting CR has been part of medical training since the 1970s [4], the implementation of ICE in physical therapy education is more recent. Defined as “a clinical education experience that occurs during an academic term in a coordinated fashion with didactic courses” [4], the proposed benefits of ICE in entry-level physical therapy training include, but are not limited to, increased trainee validation of their chosen profession, exposure to role models, and additional practice of foundational tests and measures. While ICE embodies multiple forms, an increasing presence of academically linked physical therapy studentrun free clinics (SRFCs) have emerged in the United States [5], aiming to bridge the gap between classroom and clinic, while simultaneously serving the community. Regrettably, assessments of CR during ICE within physical therapy education are sparse; the majority of investigations have been performed in other allied health fields [3,5,6]. Similarly, problem-based learning (PBL), a classroom model that creates a learner-centered environment where students solve complex clinical situations in a small-group format, has garnered significant attention [7]. Although assessments of the impact of PBL have varied, improved examination scores, social and cognitive domain performance, and self-reported CR have been noted, primarily in studies in the medicine, pharmacy, dental, nursing, and occupational therapy literature [7]. Unfortunately, longitudinal assessments of CR in physical therapy education are limited [3], particularly those examining the implementation of ICE with concurrent PBL during the final year of didactic training [8]. Consequently, the purpose of this investigation was to examine whether ICE with concurrent PBL was associated with increased self-reported CR in third-year student physical therapists (PTs). We hypothesized that ICE with concurrent PBL would be associated with improved self-reported CR in thirdyear student PTs in the United States.

A quasi-experimental nonrandomized pretest-posttest design was used with a convenience sample of 42 participating student PTs during their third and final year of didactic coursework in the Midwest of the United States. All participants completed informed consent following ethical approval granted by the university’s Institutional Review Board (Ref# 238218). Students participated in two 8-week PBL courses, with concurrent ICE throughout. The ICE stemmed from a departmental-sponsored and faculty-supervised SRFC that provided pro bono physical therapy services 2 afternoons per week to underserved individuals within the community. Clients across the lifespan were treated, including those with neurologic and orthopedic diagnoses. Third-year students were paired with an underclassman and faculty supervisor for all clinical care. Simultaneously, the students completed two 8-week PBL courses with small-group case-based sessions totaling 6 hours per week for 16 weeks. Topics included the content areas of pediatrics, geriatrics, community-based practice, and mental health. The groups were small (limited to 6 students), with a local physical therapist or faculty member as a PBL tutor. After reaching conclusions regarding each case, students completed a clinical presentation using the International Classification of Functioning, Disability, and Health framework to disseminate their findings [3].

The Self-Assessment of Clinical Reflection and Reasoning (SACRR) survey, with 26 items rated on a 5-point Likert scale, ranging from 5 (strongly agree) to 1 (strongly disagree), is a reliable and valid tool for examining students’ self-reported perceptions of CR development across multiple curricular designs in allied health training, including physical therapy [5,6,8,9]. The SACRR was reported to have an internal consistency of 0.87 pretest and 0.92 posttest using the Cronbach alpha, with a moderate Spearman rank-order correlation coefficient for test-retest reliability at 0.60 [9]. The pretest-posttest survey analyses included changes in SACRR individual item responses and overall scores (aggregate of all items) between the beginning and the end of the 16-week session. All statistical analyses were performed using IBM SPSS Statistics for Windows ver. 25.0 (IBM Corp., Armonk, NY, USA), utilizing the paired t-test for comparisons with significance accepted at P< 0.05.

Upon examining the SACRR pretest-posttest analysis for overall scores and individual items from third-year student PTs completing ICE with concurrent PBL coursework, the following results were found. The overall scores were normally distributed, demonstrating a statistically significant increase from 105.43± 7.18 to 109.40± 7.14 (P< 0.001). However, all 26 SACRR individual items demonstrated a non-normal distribution, so the Wilcoxon rank-sum test was used to analyze them. Of the 26 SACRR items, 6 demonstrated statistically significant improvements (Table 1). These items (1, 6, 11, 17, 22, and 24) clustered around 2 general themes, including decisionmaking based on experience and evidence, as well as self-reflection and reasoning. The raw data are available in Supplement 1.

This study found that self-reported CR improved in third-year student PTs after completing ICE with concurrent PBL coursework, as shown by an increased overall SACRR score and improvements on 6 individual items (Table 1). The items that showed improvement had themes associated with decision-making based on experience and evidence, as well as self-reflection and reasoning. Our results are consistent with those of Seif et al. [5], who noted improvements in SACRR scores across various allied health professionals participating in an SRFC. Similarly, Coker [6] found that 1 week of hands-on ICE was associated with elevated SACRR scores among occupational therapy students. Further, Hakim et al. [4] underscored the benefits of ICE, emphasizing that such clinical environments provide the necessary time for students to develop self-reflection and CR, while allowing academic programs to closely coordinate the clinical curriculum and to reinforce classroom concepts.

Furthermore, in contrast to investigations that only demonstrated improved SACRR performance during full-time clinical rotations [6], this study highlights enhanced self-reported CR with as little as 2 half-days of clinic per week when coupled with PBL. This fills a knowledge gap by not only examining the potential benefits of ICE on CR within physical therapy education, which is limited, but also by documenting the additional benefits of concurrent PBL, with detailed information about the implementation thereof. The positive attributes of PBL coursework were reinforced by Fan et al. [7], who noted enhanced reflective practice and CR. Moreover, the utilization of PBL within curricula is of growing prominence as national physical therapy licensure examinations have steadily increased the number of case-based clinical questions to determine entry-level readiness [10].

Although ICE and PBL have benefits, several limitations have been noted [7]. Recent findings suggest while students’ overall perception of PBL is positive, students and faculty are faced with an increased preparation time, periods of potentially inadequate discussion, and instances of complaints about group size [7]. Moreover, PBL outcomes are not consistently favorable in comparison to those of traditional teaching methods [7], especially on foundational science examinations. Likewise, while moderate evidence supports the proposal that ICE leads to improved CR and student perceptions of inter-professional attitudes [6,9], several challenges have been noted. A major concern is the ability of the ever-growing number of graduates to match with high-quality clinical sites, especially with traditional one-to-one preceptor-to-student models [4]. Additionally, quality ICE models entail significant resources, time, commitment, and energy [4]. The authors acknowledge that the ICE and PBL models face challenges, but we believe that the benefits of creating an innovative learning environment linking classroom and clinical concepts in the provision of care to underserved individuals far outweigh such difficulties.

Even though the overall SACRR scores improved, as well as those of 6 of the 26 items regarding decision-making based on experience and evidence and the promotion of self-reflection and reasoning, items associated with dealing with uncertainty and the incorporation of theory into practice showed non-significant improvements. This reinforces previous calls [1,3] for physical therapy educators to increase the explicit use of theoretical frameworks linking classroom and clinical preparation to more effectively facilitate a praxis of learning. Subsequently, investigations aimed at improving students’ ability to deal with uncertainty using theoretical frameworks to guide educational and clinical practice are warranted [2,3].

The limitations of this study include the fact that SACRR scores were self-reported, without objective measures of CR within the classroom or clinical setting. Additionally, the small sample size limits generalizability, and geographical and institutional differences in educational practice must be considered when transferring findings beyond student PTs in the Midwest of the United States. Furthermore, a more robust experimental design with randomized student assignment using a proper control group would enhance the value of this study, as the current design was quasi-experimental in nature.

This investigation measured changes in the self-reported CR of 42 student PTs completing an ICE and concurrent PBL program during their third and final year of didactic training. Improvements in the overall SACRR score, as well as the item-level analyses, are consistent with previous investigations [5,6,8,9], although prior scholars have often examined only 1 of the 2 curricular components. Future investigations are recommended to examine educational strategies to increase students’ self-reported use of theoretical frameworks and to improve their ability to cope with uncertainty. By performing longitudinal assessments of key educational domains, educators may highlight curricular designs and teaching techniques suitable for enhancing outcomes in and beyond the classroom.

## Figures and Tables

**Table 1.** SACRR pretest and posttest comparisons

SACRR items		Pretest	Posttest	P-value
**1.**	**I question how, what and why I do things in practice.**	**4.40 ± 0.77**	**4.64 ± 0.49**	**0.03**
2.	I ask myself and others questions as a way of learning.	4.62 ± 0.49	4.76 ± 0.43	0.13
3.	I don’t make judgements until I have sufficient data.	3.90 ± 0.62	4.02 ± 0.72	0.29
4.	Prior to acting, I seek various solutions.	4.07 ± 0.51	4.10 ± 0.58	0.82
5.	Regarding the outcome of proposed interventions, I try to keep an open mind.	4.29 ± 0.51	4.36 ± 0.49	0.44
**6.**	**I think in terms of comparing and contrasting information about a client’s problems and proposed solutions to them.**	**3.86 ± 0.72**	**4.36 ± 0.49**	**< 0.001**
7.	I look to theory for understanding a client’s problems and proposed solutions.	3.86 ± 0.68	3.93 ± 0.75	0.49
8.	I look to frames of reference for planning my intervention strategy.	4.02 ± 0.56	4.10 ± 0.66	0.41
9.	I use theory to understand treatment techniques.	4.02 ± 0.72	4.12 ± 0.55	0.29
10.	I try and understand clinical problems by using a variety of frames of reference.	4.17 ± 0.62	4.19 ± 0.71	0.83
**11.**	**When there is conflicting information about a clinical problem, I identify assumptions underlying the different views.**	**3.67 ± 0.69**	**3.93 ± 0.51**	**0.03**
12.	When planning intervention strategies, I ask“What if”for a variety of options.	4.14 ± 0.72	4.33 ± 0.65	0.13
13.	I ask for colleagues’ideas and viewpoints.	4.69 ± 0.47	4.69 ± 0.47	1.00
14.	I ask for the viewpoints of clients’family members.	4.02 ± 0.72	4.24 ± 0.62	0.11
15.	I cope well with change.	3.79 ± 0.65	3.76 ± 0.53	0.81
16.	I can function with uncertainty.	3.60 ± 0.80	3.69 ± 0.68	0.32
**17.**	**I regularly hypothesize about the reasons for my clients’ problems.**	**4.24 ± 0.66**	**4.52 ± 0.55**	**0.01**
18.	I must validate clinical hypotheses through my own experience.	3.83 ± 0.88	4.07 ± 0.84	0.06
19.	I clearly identify the clinical problems prior to planning interventions.	4.19 ± 0.59	4.33 ± 0.53	0.16
20.	I anticipate the sequence of events likely to result from planned interventions.	4.12 ± 0.74	4.26 ± 0.45	0.24
21.	Regarding a proposed intervention strategy, I think,“What makes it work?”	4.24 ± 0.53	4.33 ± 0.65	0.41
**22.**	**Regarding a particular intervention, I ask, “In what context would it work?”**	**3.90 ± 0.79**	**4.17 ± 0.54**	**0.04**
23.	Regarding a particular intervention with a particular client, I determine whether it worked.	4.52 ± 0.51	4.55 ± 0.50	0.80
**24.**	**I use clinical protocols for most of my treatment.**	**3.17 ± 0.88**	**3.57 ± 0.89**	**0.01**
25.	I make decisions about practice based on my experience.	4.24 ± 0.62	4.33 ± 0.53	0.29
26.	I use theory to understand intervention strategies.	3.86 ± 0.78	4.05 ± 0.62	0.12
**Total score**		**105.43 ± 7.18**	**109.40 ± 7.14**	**< 0.001**

Values are presented as mean±standard deviation. Statistical significance (P<0.05) indicated with text in bold. SACRR, Self-Assessment of Clinical Reflection and Reasoning; scale=1 (strongly disagree) to 5 (strongly agree).

## Supplementary materials

Supplement 1. Data files are available from https://doi.org/10.7910/DVN/VCFDR0.

Supplement 2. Audio recording of the abstract.
